# Opinions on Insect Consumption in Hungary

**DOI:** 10.3390/foods9121829

**Published:** 2020-12-09

**Authors:** Katalin Szendrő, Katalin Tóth, Mónika Zita Nagy

**Affiliations:** 1 Institute of Marketing and Management, Szent István University Kaposvár Campus, H-7400 Kaposvár, Hungary; 2 Institute of Regional and Agricultural Economics, Szent István University Kaposvár Campus, H-7400 Kaposvár, Hungary; toth.katalin@szie.hu; 3 Institute of Methodology, Szent István University Kaposvár Campus, H-7400 Kaposvár, Hungary; nagy.monika.zita@szie.hu

**Keywords:** insect, insect-based food, knowledge, attitude, consumption, Hungary

## Abstract

The aim of the study was to assess knowledge and opinion in the Hungarian population about the consumption of insect-based food. The questionnaire was filled in by 414 respondents. Their knowledge of edible insect consumption in different countries was average (score 4) or above on a scale of 1 (totally disagree) to 7 (totally agree). Their willingness to consume insect-based food was low, usually below average. Significantly higher scores were attained by men than women, by respondents with a university degree than those who graduated from secondary school, and the highest scores were attained by people of 30–39 age group. However, the effects of residence (town or village) and income were not significant. As Hungarians are not traditional insect consumers, there is a significant emotional response of disgust regarding food made from insects and, as insect food is not commercially available, no significant increase in insect consumption is expected in the near future.

## 1. Introduction

Meat and animal protein consumption has a long history. Animal protein, including essential amino acids, energy, and fatty acids, mainly omega-3, have all played an important role in the development of homo sapiens and its brain [1,2]. Initially, a hunter-gathering lifestyle was pursued, and later the domestication of several animal species began. In parts of the world where there was a limited supply of meat and other foods of animal origin, especially where starvation occurred, humans tried to meet their needs by eating insects [3].

The world population is growing [4], living standards are rising [5] and people are consuming more and more meat and other food products originating from animals [6]. This is experienced not only in developed countries, but also in low- and middle-income countries [7], however, available food sources are limited. At the same time, there has been strong pressure to reduce meat consumption, because of the belief that animal husbandry plays an important role in global warming, greenhouse gas emission, etc. [8,9]. It seems that it will be necessary to find alternative protein sources. Insects as a major animal group represent a high ratio of the total biomass in nature that is generally wasted [10]. Some scientists recommend the consumption of alternative foods, such as edible insects (adults, eggs, larvae, pupas, nymphs), since these could be a novel, alternative protein source [3,11]. They can be eaten raw, fried, boiled, roasted or ground at various life stages [12].

Entomophagy is an ancient food habit [13]. Nowadays the Food Organisation of the United Nations (FAO) and the United Nations (UN) suggest insects as an alternative protein source in human food and animal feed [3]. Several authors have summarized the benefits of insect production and consumption and provide a long list of benefits: less land [3], water and energy needed [3,11,14], decreased climate change, greenhouse gas emission [3,15] and ammonia [14], compared to meat production and consumption. Edible insects generally have a high nutritional value, including protein (essential amino acids), energy [3,16], healthy polyunsaturated fatty acids such as omega-3 (low ratios of n-6/n-3 fatty acids (FAs)) [3,16], micronutrients (copper, iron, magnesium, phosphorus, selenium, zinc) [3,16], and certain vitamins (riboflavin, pantothenic acid, biotin) [3,17]. Therefore, insects can play an important role in ongoing global nutritional challenges [12]. Insects (2000 edible species) are part of traditional diets for about 2 billion people in developing countries in Central and Western Africa, South East Asia, and Central and South Africa [3]. Knowledge of edible insects is inherited from generation to generation [10]. In developing countries, “wild” insects are harvested in nature [18]. However, insect consumption is unusual in western countries, although in recent years more people are trying them, and insect food is available in some countries [15]. Some insect species are farmed and sold as food for humans in Thailand, China, South Africa, the USA, the Netherlands, Belgium, France and Switzerland [18]. New Zealanders believe that insects are an environmentally sustainable food source, which could be an alternative to meat products, hence they are more receptive to insects than Europeans [19], but there is also a big difference between European countries. Nowadays, insects are also consumed in the Netherlands, Belgium, Switzerland and France. Consumers in Nordic countries showed higher acceptance for eating insects [20] than in Poland [21] or in Italy [22], which could be in connection to different food cultures [15]. Differences can also be found between North and South (closer to the traditional Mediterranean-type diet) Italy [23], and between Northern and Southern China [12]. People who live in a country with a traditional food culture may be less susceptible to accepting new products such as insects than people living in a rapidly changing food culture [24] and Italian food culture is one of the strongest in Europe [24]. Laws on using insects as food are not yet available in several countries [25]. In Europe, this legislation is necessary as insects are accepted as a novel food [26]. In Hungary there is an insect-eating group (Bugfood Foundation) [27]. However, they purchase insects from abroad. In Hungary and in most Western countries one of the main barriers is disgust and other negative aspects: they are considered dirty, disgusting and dangerous; eating insects is connected with primitive people’s practice, etc. [3,24].

Several papers have been published in Europe and elsewhere on the acceptance and rejection of edible insects [12,20,22,24,28,29,30]. Most of these have been conducted in the Netherlands and Belgium [31,32]. So far, only one article has been published on the opinions of Hungarians regarding insect consumption [33]. The aim of the present study is to get to know about Hungarian respondents’ knowledge of insect consumption and the reasons for rejection, acceptance and motivation.

## 2. Materials and Methods

### 2.1. Sampling Method

The national consumer research was conducted in Hungary in 2020. The structured questionnaire asked the respondents regarding their knowledge and their opinion on consuming insect-based food on a 1–7 scale (1 = totally disagree; 7 = totally agree; hence score of 4 is average).

Among non-probability sampling techniques, snowball sampling of data collection [34] was use. The survey was given to an initial group of respondents (those who used the Internet), selected not randomly. The survey was delivered to familiar persons via social media. After being interviewed, respondents were encouraged to locate other members of the target population whom they knew, e.g., friends, relatives and colleagues, who were willing to fill in the questionnaire. Answering was voluntary. Multiple responses were excluded since the system allowed only one response/IP address. The survey consisted of 15 structured questions. The total number of responses was 414 (Table 1). Subgroups were formed based on background information: gender, age, education background, residence and household income.

### 2.2. Statistical Analysis

Only correctly completed questionnaires were evaluated and responses were analysed among those who regularly consume meat or animal products (dairy products, eggs). The questionnaire was evaluated with one-way ANOVA using STATA/MP 15.1 software:

Y_ij_ = μ + V_i_ + e_ij_ (1)

where: μ = general mean, V_i_ = effect of the variables (i = 1–2), and e_ij_ = random error. Cross tables (for determining the relation of a variable to the background variables and other involved variables) were used in the evaluation of the questionnaires. Beside mean and standard deviation calculations, the significance of differences was tested by Tukey’s post hoc test. Moreover, f-test and *t*-test were performed to verify proven differences in variances and means, and descriptive statistical methods were employed. For background variables, those respondents whose proportion did not reach 3% were excluded from the analyses due to the low number of items.

## 3. Results

The responses were grouped by topic, and the answers to each question are discussed in descending order from the highest average score.

After evaluating the respondents’ answers to the questionnaire, the results were summarized in tables: gender differences in Table 2, the effect of age in Table 3, and the effect of educational background in Table 4. As the effect of residence (city or village) was significant only in one case and income did not affect the results in any case, these results are only mentioned in the text.

### 3.1. Knowledge about Insect Consumption

Hungary has no tradition of consuming insects and insect-based food. There are no events or campaigns to promote the consumption of insects [33], and even the distribution of insects and insect products is prohibited [35], hence whoever wants to consume such products must obtain them from abroad. Nevertheless, there are published articles on the benefits of using insect protein, mainly on the use of insects in animal feed, but also for human consumption. That is, anyone who is a regular newspaper reader, listens to the radio and watches TV programs can obtain information about the benefits of insect consumption.

- “I am aware that insects and their lava are regularly consumed in African, Asian and Latin American countries.”

People have been consuming insects since the arrival of Homo sapiens [36], but nowadays entomophagy is practiced only in less developed counties and regions. China has a long history of insect-eating. In the ancient period (Song and Ming Dynasties) people consumed ants [12]. Nowadays, insects are regularly consumed in different regions [12]; about 13% of Chinese people regularly consume insects and 55% have consumed insects in their lifetime [28]. In Zimbabwe, about 80% of the urban population consumes insects [37]. However, a decrease in the prevalence of traditional practices of entomophagy in developing countries has occurred due to the westernization of traditional diets [38].

In the present study, respondents were well informed regarding this question giving an average of 6.05 points on a scale of 1–7. No significant difference was found between the genders (Table 2). However, t-test verified differences; greater variance was found among men’s responses. The 30–39 age group had the most knowledge, while the youngest generation had the least knowledge (Table 3). Those holding a university degree also gave a higher score than those with secondary education (Table 4). Moreover, the f-test and t-test verified differences among variances and means, respectively. The effects of residence and income were not significant.

- “I am aware that edible insect food is available in several European countries.”

Respondents gave a much lower, little higher than average, score to this question (4.13). The effects of any factor (gender, age, education, place of residence and income) were not significant and all respondents gave a similar score (Table 2, Table 3 and Table 4).

Hungarian respondents’ knowledge is lower, but is in line with surveys in other countries. According to a former Hungarian survey [33], about 60% of respondents answered that they had heard about eating insects. In a German study, 80% of respondents had been aware of insect consumption in Europe [30]. High willingness to try consuming insect-made food can be found in Belgium and the Netherlands [39,40]. In Australia, 68% of respondents had heard about entomophagy, however, only 21% had previously eaten insects [41]. The ratio was higher in neo-philic consumers (79%) than neo-phobic (55%). In the future, the acceptance and availability of insects and insect food will continue to grow because some European and other countries have a general strategy to increase the acceptability of insects in food [42].

- “I think edible insect food can be made for civilized people.”

Respondents gave a little lower than the average score (3.92) for this question, which, in agreement with the previous point, means that the practice of eating insects may become more general, and not limited to a few continents and countries. Higher scores were given by men (*p* < 0.001), people aged 30–39 (*p* < 0.001), and respondents with higher education (*p* < 0.001) (Table 2, Table 3 and Table 4), however, the effect of residency and income were not significant. T-test verified differences among the means in gender and educational background.

According to results of questionnaires conducted in several countries, about 97% of respondents were aware that insects could be cooked for food [43].

- “In my opinion, edible insect food is only eaten by primitive people.”

In line with the previous question, respondents gave a very low score to this question (2.23). While no difference was found between the opinions of the genders, the oldest age group (>50 years) and those with a secondary education gave higher scores than others (*p* < 0.001 and *p* < 0.05, respectively). (Table 2, Table 3 and Table 4). T-test verified differences among the means regarding educational background. The effects of residency and income were also not significant.

In some western countries, insects are seen as primitive food, whereas some other cultures consider them to be a valuable part of the diet [44].

- “I think the silkworm drink can be nutritious.”

As the world’s silkworm rearing is concentrated in China, consumption of silkworm is traditional, and the nutritional value and health benefits of silkworm pupae [45] are known there. The question about the particular drink was not about its acceptance, but rather about the nutritional value of insects in general. Respondents certainly have some knowledge of the nutritional value of insects and their products, and therefore gave a higher score to this question than for several other questions, but still lower than average (3.45). Men, and people between ages 30 and 49, gave scores of around 4, while women and respondents between age 18–29 and above 50 years gave scores of around 3 (*p* < 0.05 and *p* < 0.001, respectively; Table 2 and Table 3). A significant difference was found also between groups of higher educated people and those who finished secondary school (Table 4). T-test verified differences among means in gender and educational background, while the effects of residency and income were not significant.

The Chinese Ministry of Health promotes silkworm pupae as a valuable protein source [12]. Comparing insect-based food in China, the highest value of willingness-to-eat was observed for the silkworm drink [28].

### 3.2. Rejection

- “I find insect food disgusting.”

Food neophobia means the tendency of the individual to avoid any unfamiliar foods [23]. In the past, this was beneficial because it could protect humans from supposedly dangerous food. Nowadays, rejection may occur of new and unusual food [46]. The origins of disgust are rooted in culture [29]. Socio-cultural factors determine the rejection of insects [28,30]. For most Western people insects are considered dirty, dangerous and disgusting, and they have an aversion to eating them [47]. The main perceived barriers are the sense of disgust arising from seeing insects and incompatibility with the local food culture [48]. Food neophobia level (low, medium, high) is closely correlated with willingness to accept insects. Food neophobia is also a barrier to the consumption of insects in Hungary, and it is higher (3.21 on a 1–5 scale) than in most Western countries [33].

This view was also supported by the current investigation. Respondents gave an average score for this question of 3.96, which shows that there is insect-phobia in Hungary, though not significant. As expected, women gave 0.79 points higher than men (*p* < 0.05, Table 2). Those with secondary education were also more dismissive than those with a university degree (*p* < 0.05, Table 3). T-test verified differences among means in gender and educational background, while the effects of age, residence and income were not significant.

### 3.3. Acceptance (Depending on the Preparation)

- “I think they can make insect food well in a restaurant.”

It is interesting that, compared to other questions, quite a high score (3.91) was given by respondents regarding insect-based dishes prepared in a restaurant. However, it can be stated that the effect of none of the examined factors was significant (Table 2, Table 3 and Table 4).

A higher level of willingness to accept insects in an ethnic restaurant was found in people with low food neophobia [29]. People go to an ethnic restaurant because they want to eat special food there. However, the question was about restaurants in general, which may indicate some confidence related to insect food.

- “I would eat food that contains processed insect products (e.g., cakes).”

The level of acceptance of food that contains processed insect products by respondents is below average (2.99). Such food is more favoured by men than women (*p* < 0.001), 30–39-year-old people than older generations (*p* < 0.001), and those with a university degree (*p* < 0.001) (Table 2, Table 3 and Table 4). T-test verified differences among means in gender and educational background, and one-way ANOVA confirmed differences among variances regarding educational background, while the effects of residency and income were not significant.

Insect food was more accepted when the consumer did not see insects in the product [28,33] or when they were present only in the list of ingredients. In these cases, consumers tend to be more open to tasting the product [22,32]. The visual form of an insect is in close connection with their acceptance. The lower the visibility, the higher the respondents’ acceptance [30]. Including insects in food products (beef stew, curry, brownies, spice cakes, tortilla chips, whole mealworms and grasshoppers crushed or in meatballs, jelly with whole cricket and jelly-based insects, chocolate chips cookie, etc.), acceptance greatly improved [30,46,48,49,50]. In an Italian study, the mean acceptability was the highest in the case of biscuits using insect flour and chocolate-coated grasshopper, and the lowest in risotto containing maggots, maggot cheese, and lollipops containing larvae [29]. People are more receptive of insects if they are mixed into a food that is currently being consumed than to eating them individually [19,51]. This may be related to the fact that, if a non-familiar food is given together with a favourite or a sweet food, its acceptance can be increased significantly.

In general, men are more receptive, more adventurous and more curious and prefer to try something new, so they are more likely to taste food made from insects than women. At the same time, women are generally more neophobic than men [28,31,47,52,53]. Women consider the following factors, taste, disgust factor, food safety, lack of familiarity and lack of perceived benefits, to be more important barriers to insect consumption than men [19]. The explanation of the differences in disgust or acceptance between genders could be due to social or cognitive rather than biological reasons [54]. The gender effect was small but significant [46]. Laureati et al. [29] and Wilkinson et al. [41] and others experienced more willingness to consume insects in males than in females. In a former Hungarian study, males were more ready to eat insects than females [33]. In the present study also, a significant difference was found between the two genders.

In general, younger people are more likely to accept new food, including that made from insects [31,52,55]. Generally, young people are more open to new things and to trying out new food [19]. In an Australian study, similar responses were received for younger and older respondents [41], however, in general the readiness to eat insects was stronger among young respondents than among older ones (23,29,31]. In another study, 6–11 years old children were more interested in insect products, as they were likely to focus on the external appearance of the product and not on the insect content [43]. The reverse is also true; neophobia is more common among the elderly [19,56]. Older people have more and stronger barriers than the younger generation [19]. The role of social norms is a more important predictor of the dietary intentions of adults than that of the young [48]. The older respondents want to know more about the food they consume. The older individuals are, the more disgusted they are by unknown foods [54]. China is an exception since the elderly there prefer to eat insects because during the Great China Famine insects were the main protein source [12]. The results of the present study are only partially consistent with the literature data, because the highest scores were given by respondents between the 30–39 age group.

Laureati et al. [29] reported that more university students and staff (higher level of education) accepted insects as food than people outside the university. People with higher education have more knowledge, so they are more positive about novelty than less educated people. For this question, and in general, the same can be seen in the present study.

- “I would like to taste a whole insect (e.g., cricket, locust) fried well.”

Insect-based food is less preferred when the whole insects are visible [22,28]. The willingness to eat food containing insects is higher when they are mixed in flour form (e.g., into a pizza) or they are covered with sweets such as chocolate [42,43,52].

In the present study, fewer respondents would consume the whole insect (2.54) than if the food contained processed insect (2.99). The preference was higher by 1.32 points among men than women (*p* < 0.001; Table 2). Higher scores were given by people aged 30–39 years than the youngest (18–29 years) and the oldest (> 50 years) generations (*p* < 0.05; Table 3), and by higher educated respondents than those who finished secondary school (*p* = 0.01; Table 4). F-test and t-test verified differences among variances and means, respectively, regarding gender and educational background.

- “I’d even make food at home that contains insect flour.”

According to the average score (2.38), it seems that homemade food is less preferred than that containing processed insects (see the 2.99 score given to that question). It can be assumed that the respondents were also influenced by the fact that they had to work with insect flour and had to mix it into the food, although, in both cases, there was a similarity in the scores given by men and women (*p* < 0.001; Table 2). Based on age, a significant difference was found between the ages of 30–39 years, and over 50 years of age, higher scores given by the younger respondents (Table 3). Significantly higher scores were also given by higher educated people than those who finished secondary school (Table 4). T-test and one-way ANOVA verified differences among variances and means, respectively, regarding gender and educational background. The effects of residence and income were not significant.

- “I would eat insect products if special attention was not given to the packaging; it would only be included in the ingredients.”

Among the effect of preparation, this question received the lowest scores (2.21). The value was not modified by any examined factors (Table 2, Table 3 and Table 4). There seems to be a more negative effect if the packaging does not draw enough attention to the fact that the product contains insect flour.

### 3.4. Motivation

- “If it turned out that my boyfriend/girlfriend/family member was eating insect-based food, I would also try it.”

It seems that a close family member or friend may influence the acceptance of food made from insects. The average score for this question was a little higher than the average (3.19). This was particularly important for men, with a 1.26 difference between the two genders (*p* < 0.001; Table 2). This effect was also significant in respondents aged 30–39 and who were higher educated, while it did not play an important role for the elderly (> 50 years) and for those who finished secondary school (Table 3 and Table 4). T-test and one-way ANOVA verified differences among means in gender and educational background.

There are also references in the literature to the role of family members’ and friends ’opinions. Insect acceptance was favoured if family members, and even more if friends, recommended it [46]. According to respondents, if a chef or a celebrity proposed it, they would be more to likely eat insects. At the same time, they would also prefer to eat insects if recommended by a friend [43].

- “I wouldn’t refuse insect food as a guest.”

Politeness can also play a role. If respondents were guests, they would show moderate acceptance (3.01). Gender played a role in this; men’s attitudes would be better than women’s (Table 2). While the effect of age was not significant (Table 3), those with higher education would accept insect food more positively as guests than those with secondary school education (*p* < 0.05; Table 4). T-test and one-way ANOVA confirmed differences among means in gender and educational background.

- “I would only taste insect food abroad (e.g., in the Far East).”

The low acceptance of insect consumption is shown by the fact that respondents would not like to eat such food even abroad, where it is commonly consumed. The average score (2.09) was not modified significantly by any factor (gender, age, education, residence or income; Table 2, Table 3 and Table 4).

### 3.5. Attitude

- “I think insect food is exotic (strange/interesting).”

Respondents gave a high score to insect consumption in Asia, South America and Africa, and these places are generally considered exotic, so insect food was associated, with an average score of 4.31. Higher scores were given by men than women (*p* < 0.001; Table 2), respondents between age 30 and 49 than people above 50 years (*p* < 0.05; Table 3), higher educated respondents than those who finished secondary school (*p* < 0.05; Table 4), and the group living in a village than that living in an urban area (*p*<0.05). T-test also verified differences among means in gender, educational background and residency. Income was the only factor that did not affect the score. Eating insects is still considered exotic in Hungary [33].

- “I would like to taste food made from insects.”

Consumption of insect food was rated lower than average by respondents (2.85). Men gave 1.17 higher points than women (*p* < 0.001; Table 2), and there was a difference of 1.28 points between the ages of 30–39 and those over 50 years of age (*p* < 0.001; Table 3). There was a smaller difference between university and secondary school graduates (*p* < 0.05; Table 4). However, these higher acceptance values are also lower than the average level. F-test and t-test verified differences among variances and means, respectively, regarding gender and educational background. Residence and income impact were not significant.

The acceptance of insect consumption in Hungary is low [33]. The proportion of those who would accept insect food instead of meat is very low.

In an Italian study, 16.7% and 4.4% of respondents agreed and strongly agreed with incorporating insects into different food [29].

- “I am interested in insect food.”

In general, insects are unfamiliar in most Western countries [24,26]. However, as stated earlier, in addition to traditional insect-consuming countries [12,28,32], the acceptance of insect food is significantly higher mainly in the Netherlands [49,53] and Belgium [57], but also in some other countries [28,29,30,43,46]. It is less accepted in Central Europe than in Northern Europe [20], and mainly after previous tasting and presenting insects in more familiar forms. In a former Hungarian survey low willingness to sample insect-based food was registered [33].

The above was confirmed by the present study. Interest was much lower than average in insect food (2.52). The difference between the genders (*p* < 0.001; Table 2), age categories (*p* < 0.05; Table 3), and education (*p* < 0.05; Table 4) was slightly smaller than for the previous question. T-test and one-way ANOVA verified differences among variances and means, respectively, regarding gender and educational background. The effects of residence and income did not significantly modify the given scores. Even in the groups with higher scores, only average interest could be measured.

- “I find insect food to be delicious.”

The lowest scores were given for this response (2.25). Higher scores were given by men than women by 0.99 points (*p* < 0.001; Table 2), by respondents between 30–39 than 18–29 years of age (*p* < 0.05; Table 3), and by people with a university degree than finished secondary school (*p* < 0.05; Table 4), but even these higher scores were below average. T-test verified differences among means in gender and educational background, while f-test confirmed differences among variances regarding educational background. Respondents’ opinions were not influenced by residence or income.

## 4. Conclusions

As there is no tradition of insect consumption in Hungary, this food is not commercially available and there are no initiatives to encourage consumption, so naturally the acceptance of insects and insect food is low and it is rejected by more than average. According to the survey, respondents are well informed about the benefits of consuming insects and using them as food. Hungary can be a model for countries that are less fond of insects and insect foods. There is also a tendency for men and graduates to prefer insect foods by more than average, while among the age groups, 30–39-year old gave the highest scores. Based on the results, it is not expected that insect consumption will increase significantly in the near future in Hungary.

## Figures and Tables

**Table 1:** Distribution of the sample.

**Description**	*n* ^1^	%
Total respondents	414	100
**Gender**		
Female	271	65.5
Male	143	34.5
**Age**		
18–29	98	23.7
30–39	99	23.9
40–49	100	24.2
>50	117	28.3
**Education background**		
Secondary school	101	24.4
College, university	313	75.6
**Residence**		
City	318	76.8
Village	96	23.2
**Household income**		
Just enough, but cannot set any money aside	63	15.2
Live well but can only set little money aside	205	49.5
Live very well and with a high enough income to set money aside	120	29.0
No answer/Don’t know	26	6.3

^1^ Number of items.

**Table 2:** Effect of gender of respondents on opinions on insect-based foods and their consumption.

	Gender				*p*
	Women		Men		
	Mean	SD	Mean	SD	
**Knowledge about insect consumption**					
I am aware that insects and their larvae are regularly consumed in some African, Asian and Latin American countries.	6.16	1.39	5.85	1.71	0.068
I am aware that insect-based food is available in some European countries.	4.04	2.24	4.29	2.18	0.310
I think insect food can be made for civilized people.	3.62	2.16	4.51	2.18	<0.001
In my opinion, insect food is only eaten by primitive people.	2.24	1.75	2.21	1.85	0.860
I think a silkworm drink can be nutritious.	3.19	2.11	3.95	2.28	0.003
**Rejection**					
I find food made from insects disgusting.	4.23	2.37	3.44	2.22	0.002
**Acceptance (depending on the preparation)**					
I think insect-based food is prepared well in a restaurant.	3.78	2.03	4.16	2.02	0.100
I would eat food that contains processed insect products (e.g., cake).	2.60	2.06	3.74	2.34	<0.001
I would taste whole insects (e.g., cricket or locust) fried well.	2.09	1.84	3.41	2.37	<0.001
I would even cook food at home that contains insect flour.	1.98	1.77	3.15	2.23	<0.001
I would eat an insect product if special attention was not given to the packaging, it would only be included in the ingredients.	2.21	1.8	2.21	1.71	0.995
**Motivation**					
If it turned out that my boyfriend/girlfriend/family member was eating insect-based food, I would also taste it.	2.75	2.05	4.01	2.2	<0.001
As a guest, I would not refuse insect-based food.	2.74	2.15	3.51	2.32	<0.001
I would only taste insect-based food abroad (e.g., in the Far East).	2.11	1.66	2.06	1.55	0.770
**Attitude**					
I think insect-based food is exotic (strange/interesting).	4.14	2.33	4.62	2.20	<0.001
I would like to taste food made from insects.	2.45	1.98	3.62	2.29	<0.001
I am interested in insect-based foods.	2.15	1.83	3.22	2.19	<0.001
I believe insect-based food to be delicious.	1.91	1.59	2.90	2.08	<0.001

**Table 3:** Effect of age of respondents on opinions on insect-based food and their consumption.

Knowledge about Insect Consumption	Age, Years								*p*
	18–29		30–39		40–49		>50		
	Mean	SD	Mean	SD	Mean	SD	Mean	SD	
I am aware that insects and their larvae are regularly consumed in some African, Asian and Latin American countries	5.79 ^a^	1.65	6.34 ^b^	1.26	6.28 ^ab^	1.35	5.82 ^ab^	1.66	0.010
I am aware that insect-based food is available in some European countries.	3.85	2.13	4.30	2.10	2.22	2.33	4.14	2.31	0.561
I think insect food can be made for civilized people.	3.93 ^ab^	2.07	4.53 ^b^	2.24	4.08 ^ab^	2.26	3.32 ^a^	2.12	0.001
In my opinion, insect food is only eaten by primitive people.	1.84 ^a^	1.49	1.94 ^a^	1.62	2.13 ^a^	1.70	2.92 ^b^	2.03	<0.001
I think a silkworm drink can be nutritious.	2.97 ^a^	2.05	4.10 ^c^	2.2	4.05 ^bc^	2.22	2.85 ^a^	2.05	<0.001
**Rejection**									
I find food made from insects disgusting.	4.23	2.24	3.86	2.4	3.94	2.91	3.82	2.38	0.610
**Acceptance (depending on the preparation)**									
I think insect-based food is prepared well in a restaurant.	3.86	1.89	4.10	2.06	4.15	1.91	3.54	2.17	0.153
I would eat food that contains processed insect products (e.g., cake).	3.05 ^ab^	2.13	3.78 ^b^	2.46	2.93 ^a^	2.17	2.40 ^a^	1.99	<0.001
I would taste whole insects (e.g., cricket or locust) fried well.	2.34 ^a^	1.93	3.20 ^b^	2.44	2.55^ab^	2.05	2.17 ^a^	2.0	0.003
I would even cook food at home that contains insect flour.	2.31 ^ab^	1.97	3.04 ^b^	2.27	2.44 ^ab^	2.06	1.85 ^a^	1.61	<0.001
I would eat an insect product if special attention were not given to the packaging, it would only be included in the ingredients.	2.54 ^b^	1.96	2.43 ^ab^	1.97	2.03 ^ab^	1.62	1.89 ^a^	1.45	0.026
**Motivation**									
If it turned out that my boyfriend/girlfriend/family member was eating insect-based food, I would also taste it.	3.27 ^ab^	2.09	3.75 ^b^	2.34	3.17 ^ab^	2.15	2.65 ^a^	2.06	0.004
As a guest, I would not refuse insect-based food.	2.79	2.11	3.48	2.44	2.84	2.1	2.91	2.24	0.117
I would only taste insect-based food abroad (e.g., in the Far East).	2.29	1.72	2.16	1.69	2.17	1.58	1.81	1.49	0.152
**Attitude**									
I think insect-based food is exotic (strange/interesting).	4.03 ^ab^	2.22	4.78 ^b^	2.27	4.65 ^b^	2.26	3.82 ^a^	2.31	0.005
I would like to taste food made from insects.	2.83 ^ab^	2.04	3.58 ^b^	2.35	2.83 ^ab^	2.21	2.30 ^a^	1.89	<0.001
I am interested in insect-based food.	2.41 ^a^	1.85	3.15 ^b^	2.24	2.48 ^ab^	2.04	2.13 ^a^	1.86	0.002
I believe insect-based food to be delicious.	1.82 ^a^	1.47	2.76 ^b^	2.13	2.34 ^ab^	1.7	2.13 ^ab^	1.86	0.019

^a,b,c^ means where different superscripts differ (*p* < 0.05).

**Table 4:** Effect of educational background of respondents on opinions on insect-based food and its consumption.

	Educational Background				
	University Degree		Secondary School		*p*
	Mean	SD	Mean	SD	
**Knowledge about insect consumption**					
I am aware that insects and their larvae are regularly consumed in some African, Asian and Latin American countries	6.17	1.35	5.67	1.89	0.017
I am aware that insect-based food is available in some European countries.	4.16	2.23	4.04	2.21	0.660
I think insect food can be made for civilized people	4.17	2.18	3.25	2.17	<0.001
In my opinion, insect food is only eaten by primitive people.	2.09	1.69	2.63	1.98	0.009
I think a silkworm drink can be nutritious.	3.61	2.22	3.01	2.08	0.030
**Rejection**					
I find food made from insects disgusting.	3.79	2.35	4.48	2.28	0.014
**Acceptance (depending on the preparation)**					
I think insect-based food is prepared well in a restaurant.	4.00	2.02	3.65	2.07	0.160
I would eat food that contains processed insect products (e.g., cake).	3.20	2.3	2.40	1.89	<0.001
I would taste whole insects (e.g., cricket or locust) fried well.	2.69	2.21	2.11	1.82	0.010
I would even cook food at home that contains insect flour.	2.56	2.13	1.85	1.49	0.003
I would eat an insect product if special attention were not given to the packaging, it would only be included in the ingredients.	2.19	1.76	2.26	1.79	0.730
**Motivation**					
If it turned out that my boyfriend/girlfriend/family member was eating insect-based food, I would also taste it.	3.37	2.24	2.61	1.91	0.003
As a guest, I would not refuse insect-based food.	3.16	2.28	2.49	2.04	0.011
I would only taste insect-based food abroad (e.g., in the Far East).	2.12	1.66	2.00	1.52	0.520
**Attitude**					
I think insect-based food is exotic (strange/interesting).	4.49	2.23	3.72	2.41	0.004
I would like to taste food made from insects.	3.03	2.23	2.33	1.85	0.002
I am interested in insect-based food.	2.67	2.1	2.07	1.71	0.004
I believe insect-based food to be delicious.	2.43	1.90	1.83	1.59	0.009
